# Person-centered care: preferences and predictors in speech-language pathology and audiology practitioners

**DOI:** 10.3389/fpsyg.2023.1162588

**Published:** 2023-06-30

**Authors:** Faheema Mahomed-Asmail, Vera-Genevey Hlayisi, Karin Joubert, Louise Anne Metcalfe, Marien Alet Graham, De Wet Swanepoel

**Affiliations:** ^1^Department of Speech-Language Pathology and Audiology, University of Pretoria, Pretoria, South Africa; ^2^Division of Communication Science and Disorders, Department of Health and Rehabilitation Sciences, Faculty of Health Sciences, University of Cape Town, Cape Town, South Africa; ^3^Department of Audiology, University of the Witwatersrand, Johannesburg, South Africa; ^4^Department of Science, Mathematics and Technology Education, University of Pretoria, Pretoria, South Africa; ^5^Ear Science Institute Australia, Subiaco, WA, Australia; ^6^Department of Otolaryngology-Head and Neck Surgery, University of Colorado School of Medicine, Aurora, CO, United States

**Keywords:** person-centeredness, interpersonal attributes, person-centered care, PCC, preferences, predictors, audiology, speech-language pathology

## Abstract

**Introduction:**

Increasingly person-centered care (PCC) is being recognized as an important aspect of speech-language pathology and audiology (SLP/A) service delivery. This study aimed to (i) identify preferences toward PCC; (ii) determine predictors of these preferences; and (iii) describe the understanding and views of PCC among SLP/A in South Africa.

**Methods:**

A mixed-method design was followed utilizing an online survey and four focus group discussions. The survey included demographic questions, the modified Patient-Practitioner Orientation Scale (mPPOS), the Ten-Item-Personality-Inventory (TIPI) and an open-ended question. The focus group discussions included prompting questions which facilitated an open-ended discussion.

**Results:**

A total of 91 practitioners (39.6% speech-language pathologists) completed the online survey, with nine (44.4% audiologists) participating in the focus group discussions. A high preference toward PCC was noted, with a total mean mPPOS score of 4.6 (0.6 SD). Quantile regression analysis revealed four predictors (age, home language, sector, and personality trait openness) associated with PCC preferences. Three main categories emerged from the open-ended question and focus group discussions: (i) Positive experiences with PCC; (ii) restrictions toward PCC, and (iii) PCC exposure.

**Discussion:**

Positive (age and personality trait openness) and negative (home language and sector of employment) predictors toward PCC exist among speech-language pathologists and audiologists, with an overall general preference toward PCC. Practitioners experience facilitators and barriers toward implementing PCC including the extent of personal experiences, available resources and tools as well as workplace culture. These aspects require further investigation.

## 1. Introduction

The term patient-centered care (PCC) was introduced nearly six decades ago in psychological counseling literature where PCC theory was developed (Rogers, 1951). PCC refers to inclusive care that is centered around the needs of the patient and embodies characteristics of holistic care. Key tenets include a biopsychosocial approach and power-balanced relationships between patients and health professionals (Mead and Bower, 2000; Grenness et al., 2014). Patient-centeredness is advocated in health and rehabilitation service delivery (World Health Organization, 2021) and is important in management of chronic health conditions, including communication disorders (Grenness et al., 2014).

### 1.1. Patient-centered care vs. person-centered care

Over the past two decades a variety of terms have been used interchangeably in medical healthcare literature to name the concept, some of which include patient-, person-, client-, individual-centered care (Leplege et al., 2007). The terms patient-centered and person-centered care are the most commonly used and often synonymously. However, with the rising interest and understanding of what patient-centeredness means, scholars have argued that there is a conceptual difference between the terms (Starfield, 2011; Ekman et al., 2012; Lines et al., 2015). The crux of the difference is in understanding and acknowledging that when referring to the person as a patient, there is still an inadvertent focus on the disease and its impact on the body rather than the person’s needs (Entwistle et al., 2010). Lines et al. (2015) found that changing the language around the concept works as a powerful way to recognize that people are more than their diseases. Over time, there has been a shift from the term patient-centered care to person-centered care in efforts to semantically change focus onto the uniqueness and holistic nature of the person (Santana et al., 2018). Thus in this paper, the term person-centered care (PCC) will be used.

### 1.2. PCC in speech-language pathology and audiology

Growing evidence suggests that implementing PCC may address the holistic needs of the person, eliciting a greater adherence to treatment and result in overall increased satisfaction (Rogers, 1951; Mead and Bower, 2000; Meyer et al., 2017). Moreover, participation and involvement of persons in their healthcare as proposed by person-centeredness are in line with ethical practice principles of autonomy encouraging shared power and responsibility between patients and healthcare providers in decision making (Leplege et al., 2007; Håkansson Eklund et al., 2019).

Currently, there is no standardized measurement for practice of PCC in clinical settings, however, a recent literature review has recommended using the Patient-Practitioner Orientation Scale (PPOS) to determine practitioners’ orientation and preferences toward PCC (Bejarano et al., 2022). Furthermore, several studies have utilized the PPOS to assess health professional’s preference for person-centeredness in service provision (Krupat et al., 2000; Laplante-Lévesque et al., 2014; Manchaiah et al., 2014) enabling comparability across disciplines (Bejarano et al., 2022).

In contrast with many other disciplines within healthcare, few studies have investigated the preference and orientation of speech-language pathologists and audiologists to PCC (Laplante-Lévesque et al., 2014; Manchaiah et al., 2014, 2017; Bellon-Harn et al., 2017). A survey of 663 Australian audiologists found that the majority showed a preference toward PCC with age, gender, work experience and sector of work described as factors influencing person-centeredness (Laplante-Lévesque et al., 2014). Contrary to these findings in the United States, Bellon-Harn et al. (2017) surveyed 102 speech-language pathologists and found that person-centered preferences were not influenced by age and years of experience. Another study which polled a sample of audiologists from across India, Portugal and Iran found a positive preference for PCC, suggesting that an orientation toward PCC was present across different cultures and contexts (Manchaiah et al., 2014).

The influence of interpersonal attributes such as home language, reason behind career choice, one’s personality trait on person-centered preferences has not been previously investigated in the field of Speech-Language Pathology and/or Audiology (SLP/A). A Swedish study on nursing staff showed that health professionals’ personalities have a significant impact on their perceptions of the process as well as engagement in PCC (Elfstrand Corlin et al., 2017). Understanding how interpersonal attributes are linked to the way health professionals interact adds a new perspective on how to implement PCC (Elfstrand Corlin et al., 2017). Therefore, the current study aimed to (i) identify preferences toward PCC; (ii) determine predictors of these preferences; and (iii) describe the understanding and views of PCC among SLP/A.

## 2. Materials and methods

### 2.1. Ethical considerations

Institutional review board (IRB) approval was granted by the Research Ethics Committee Faculty of Humanities, University of Pretoria (HUM031/0921). Participants were provided with detailed information about the study and informed consent was obtained from all the participants before they were able to participate in both phases of the study.

### 2.2. Study design and participants

The study employed a two phase mixed-method design (Wisdom and Creswell, 2013) with a post-positivist (quantitative) stance supplemented with qualitative data to address the research question to a greater extent (Onwuegbuzie et al., 2009). For phase one, the quantitative component constituted an e-survey (Supplementary Content 1) distributed to practitioners providing SLP/A services in South Africa. The e-survey was distributed through online social media platforms (Facebook™, LinkedIn™, WhatsApp™), through associations (South African Speech Language and Hearing Association, South African Association of Audiologists) and by forwarding to colleagues and collaborators practising in the field of SLP/A. The qualitative component (phase two) constituted four focus group discussions which included participants from phase one who were keen and willing to participate. Additional consent was sought prior to participants being included in phase two.

### 2.3. Procedures

The e-survey (Supplementary Content 1) was made available to participants using Qualtrics (Provo, UT) during June and July 2022. The survey consisted of biographic information, the Ten-Item-Personality-Inventory (TIPI) (Gosling et al., 2003), a modified version of the Patient-Practitioner Orientation Scale (mPPOS) (Laplante-Lévesque et al., 2014) and one open-ended question.

Four virtual focus group discussions (three to four practitioners in each group) took place over 2 weeks after the conclusion of the e-survey. Those who participated in the virtual focus group discussions were compensated for data used with a 1GB data voucher.

### 2.4. Data collection materials

The first section of the survey consisted of biographic information which included age, gender, sector, home language, reason behind career choice (Supplementary Content 1). This was followed by the TIPI which was developed to measure the five core personality traits (Gosling et al., 2003). These five core personality traits include (i) emotional stability characterized by sadness, anxiety, and neuroticism; (ii) extraversion characterized by excitability, sociability, assertiveness, and high amounts of emotional expressiveness; (iii) openness to experience featuring characteristics such as a broad range of interests and creativity; (iv) agreeableness attributes such as trust, altruism, affection, and other prosocial behaviors, and (v) conscientiousness includes good impulse control and goal-directed behaviors (Cherry, 2022).

To measure each of the five personality traits (Extraversion, Agreeableness, Conscientiousness, Emotional Stability, Openness to Experience), the TIPI (Supplementary Content 1) includes two items with opposing traits (one positive and one negative) across a 10-item scale using a 7-point Likert type scale (1 = disagree strongly, 7 = agree strongly) and takes approximately 1 min to complete (Gosling et al., 2003). The test–retest reliability of the TIPI was assessed by its developers (Gosling et al., 2003) by having the same participants assessed again 6 weeks after the initial assessment and they reported the mean correlation of 0.72 to be “substantial” (p. 518). Throughout the years, the TIPI has been used in many different countries and contexts, in its original English version and translated versions, and the psychometric properties of it has been examined using various measures (e.g., test–retest reliability, internal consistency, validity (convergent, discriminant, and content), internal factor structure (exploratory and confirmatory factor analysis), and correlations between peer- and self-ratings). Overall, it has been reported to have adequate psychometric properties for the original English version (e.g., Ehrhart et al., 2009), and for the translated versions (e.g., Muck et al., 2007; Hofmans et al., 2008 [Dutch]; [Japanese]; Romero et al., 2012 [Spanish]; Storme et al., 2016 [French]). (Hanif, 2018 [Indonesian]; Nunes et al., 2018 [Portuguese]; Azkhosh et al., 2019 [Persian]; Shi et al., 2022 [Chinese]; [German]). For the TIPI scoring, the average score is taken for each of the five sub-dimensions. The personality trait belonging to the sub-dimension in which the individual gets the highest score is their primary personality trait.

The PPOS, developed by Krupat et al. (1999), is a measure of an individual’s preference toward person-centeredness and is made up of 18 questions split into two subscales, caring and sharing (both have 9 items each). The sharing subscale reflects the extent to which the respondent believes that clients desire information and should be part of the decision-making process (e.g., clients should be treated as if they were partners with the clinician, equal in power and status). The caring subscale reflects the extent to which the respondent sees the client’s expectations, feelings, and life circumstances as critical elements in the treatment process (e.g., a treatment plan cannot succeed if it is in conflict with a client’s lifestyle or values). The items are scored on a six-point Likert scale (1 = strongly agree; 6 = strongly disagree). Throughout the years, the PPOS has been used in many different countries and contexts, in its original English version (Krupat et al., 1999), and translated versions (e.g., Mudiyanse et al., 2015 [Sinala]; Paul-Savoie et al., 2015 [French]; Perestelo-Pérez et al., 2021 [Spanish]), and the psychometric properties of it has been examined using various measures [e.g., test–retest reliability, Raykov’s composite reliability, internal consistency, validity (construct, convergent), internal factor structure (exploratory and confirmatory factor analysis), item-to-scale and inter-item correlations].

For the purpose of this study, the PPOS used by Laplante-Lévesque et al. (2014) was modified to include terminology that suited both fields of SLP/A (i.e. “Audiologist” replaced by “Clinician”, “audiological condition” replaced by “communication condition”, “audiological information” replaced by “communication impairment information”, “hearing test” replaced by “assessment”).

The mPPOS was followed by a single open-ended question: “We are interested in knowing what your personal opinion and views are about person-centered care. Please write as much as you would like in the space available below.” The survey concluded with an invitation for participants willing to participate in phase two of the study to leave their email addresses.

For phase two of the study, a sub-group of nine participants participated in one of four focus group discussions. To ensure consistency, and to avoid leading remarks and a biased approach, a topic guide was developed (Supplementary Content 2). It included open-ended and follow-up probing questions regarding participants’ understanding of PCC, its benefits and restrictions and their exposure to PCC. A research assistant facilitated the online, synchronous focus groups over MSTeams™. All focus groups were video-recorded and transcribed verbatim, whilst accounting for body language, e.g., nodding (Watermeyer et al., 2012).

### 2.5. Data analysis

For the quantitative data, the Statistical Package for Social Sciences (SPSS) version 27 and a 5% level of significance were used for all statistical analyses. The Shapiro-Wilk test was used to test for normality of the scales, and since all the *p*-values were less than 0.05, the data differed significantly from normal, and, accordingly, non-parametric tests were used. Furthermore, the median (*Md*) and interquartile range (*IQR*) are reported (Supplementary Content 3) along with the mean (*M*) and the standard deviation (*SD*) due to the non-normality. The Mann-Whitney test (*Z*_MW_) was used to determine significant differences between the scores of audiologists and speech-language pathologists across TIPI and mPPOS scales. The Wilcoxon signed rank test (*Z*_WSR_) was used to determine the significant difference between the mPPOS subscales. Multiple linear regression analysis have many assumptions that must be met; one being that the residual terms be normally distributed which the Shapiro-Wilk test showed was not the case. Since not all the assumptions of multiple linear regression were met, a more robust type of regression, specifically, quantile regression, was used for regression analyses. Three regression models were built for the mPPOS total, sharing and caring subscales, respectively. For quantile regression models, we used the mean absolute error (MAE) as a measure of the quality of the models and one would like to see a reduction in percentage error from the null model (model with no predictors) to the final model (model with only significant predictors) which was the case for all three models. Robust quantile regression models were built with the following 12 predictors: participant age (continuous variable); ethnicity (three categories, “*Asian and Coloured*,” “*African*” and “*White*” with the latter as benchmark), participant home language (two categories, “*All official languages of SA except English*” and “*English*” with the latter as benchmark); practicing as (three categories, “*SLP/A*,” “*audiologist*,” *“speech-language pathologist*” with the latter as benchmark); sector of work (three categories, “*academia*,” “*private*” and “*public*” with the latter as a benchmark), population served (four categories, “*0–5*,” “*6–18*,” “*19–65*” and “> *65*” years old, with “*6–18*” years as a benchmark), most influential reason behind pursuing a career (four categories, “*career opportunities and expand knowledge base*,” “*Family members with a communication or hearing disorder*,” “*aptitude test*” and “*to help others*” with the latter as a benchmark) and the five TIPI personality traits (continuous variables). Before data collection, the required minimum sample size (nmin), for each of these statistical tests, was computed in order to know what sample size to aim for. For the ZMW and ZWSR tests, G*Power software version 3.1.9.4 (Faul et al., 2007) was used with the level of significance (0.05), minimum acceptable statistical power (0.8) and effect size (ES). For the latter, a medium to large ES (Téllez et al., 2015) was considered, as many researchers have suggested that it is unnecessary to obtain the nmin required for detecting small ES, as finding a statistically significant result for a small ES may have statistical significance (*p* < 0.05), but not real-world or practical significance (Baicus and Caraiola, 2009; Peeters, 2016). For the ZMW test, recommendations for nmin ranged from 28 (to detect large ES) to 66 (to detect medium ES). For the ZWSR test, recommendations for nmin ranged from 10 (to detect large ES) to 23 (to detect medium ES). For regression, literature has different suggestions for nmin ranging from a minimum of 5 observations per predictor, to suggestions such as nmin > 50 + p, where p denotes the number of predictors (Jenkins and Quintana-Ascencio, 2020). Since there were 12 predictors considered, the nmin ranged from nmin = 5 × 11 = 55 and nmin > 50 + 12 = 62. Thus, with a final sample size of 91, all the necessary requirements were met to ensure an adequate level of statistical power.

For the qualitative data, inductive thematic analysis using content analysis was utilized to identify themes within the data (Braun and Clarke, 2006). All transcripts were anonymized with data grouped for thematic analysis from the open-ended questions (*n* = 65) and the focus group discussions (*n* = 9). After becoming familiar with the data, two researchers (LM, FM-A) independently identified themes and coded the data. Frequency counts (#) were determined in order to support the analysis process by identifying the prominent themes with examples of meaning units captured in order to aid interpretation. If discrepancies arose they were resolved through discussion. All authors were given the opportunity to review the coded data and no changes were recommended. Finally, the categories and patterns identified were linked to the research aim to draw conclusions.

### 2.6. Quality criteria

Typically, Cronbach’s alpha is used to establish reliability for scales, however, it is well-known that Cronbach’s alpha increases as the number of items on the scale increases, as such, Cronbach’s alpha tends to perform poorly when a scale consists of only a few items. Accordingly, for scales with less than ten items, researchers (see, e.g., Pallant, 2020) have advocated that the inter-item correlation (if the scale has two items) and the mean inter-item correlation (if the scale has more than two items) be used to establish reliability. In this study, for scales with 10 or more items (the mPPOS total scale), Cronbach’s alpha was use to establish reliability, whereas, for scales with less than 10 items Cronbach’s alpha was not used (as argued previously), and, instead, the robust Spearman correlation (rs) was used to compute the inter-item correlations to establish reliability; the notation rs is used to denote an inter-item correlation with values above 0.1 indicating reliability (Pallant, 2020). For the TIPI scales (consisting of two items each), the rs values ranged from 0.216 to 0.673 with *p*-values ranging from *p* < 0.001 to 0.040, establishing reliability of all TIPI scales (Supplementary Content 4). For the mPPOS sub-scales, the mean inter-item correlation was acceptable for the sharing subscale (mean rs = 0.275; approx. three-quarters of *p*-values < 0.05) and caring subscale (mean rs = 0.204; approx. half of *p*-values < 0.05) (Supplementary Content 4) after items 10 and 17 of the caring subscale were removed as they did not correlate significantly with any of the other items on the scale. Accordingly, to establish the reliability of the mPPOS scale, items 10 and 17 were removed from all analyses providing an acceptable Cronbach’s alpha (0.837) coefficient for mPPOS total scale.

## 3. Results

A total of 91 practitioners with a mean age of 34.4 years (11.5 SD) participated in this study with the majority (39.6%) practising as speech-language pathologists. A large portion of the participants (44.0%) were employed in the public health care sector. Participants’ years of experience ranged between one to 47 years (Md = 8.0; 11.5 SD), with services provided to clients varying across age categories (Table 1). When asked about the reason behind their career choice, close to half (49.5%) indicated that their choice was a result of wanting “to help others.”

**TABLE 1:** Demographics of participants (*n* = 91).

Demographic details	Statistic
Age in years (*M*, *SD*)	34.4 (11.5)
**Gender *n* (%)**	
Female	87 (95.6)
Male	3 (3.3)
Other	1 (1.1)
**Ethnicity *n* (%)**	
African	7 (7.7)
Asian and/or colored	14 (15.4)
White	70 (76.9)
**Home language *n* (%)**	
English	45 (49.5)
Other South African Languages (excluding English)	46 (50.5)
**Profession *n* (%)**	
Audiologist	35 (38.5)
Speech-language pathologist	36 (39.6)
Speech-language pathologist and audiologist^b^	18 (19.8)
**Reason for choice of study *n* (%)**	
My aptitude test results	18 (19.8)
Family members with a communication or hearing disorder	15 (16.5)
Career opportunities	5 (5.5)
Expand knowledge base	3 (3.3)
To help others	45 (49.5)
Other	5 (5.5)
**Practising sector *n* (%)**	
Private	36 (39.6)
Public	40 (44.0)
Academic	11 (12.1)
Other	4 (4.4)
**Population served^a^ *n* (%)**	
0 to 5 years old	64 (24.7)
6 to 18 years old	75 (29.0)
19 to 65 years old	63 (24.3)
>65 years old	57 (22.0)

^a^Participants could select more than one option.

^b^Participants were practising as both speech-language pathologists and audiologists.

### 3.1. Preferences toward PCC

A mean score of 4.6 (0.6 SD) was obtained for the total mPPOS with a significant difference between the caring (4.5, 1.0 SD) and sharing (4.7, 0.7 SD) subscales, with the caring being significantly lower (*Z*_WSR_ = −2.778; *p* = 0.005) (Table 2). A significant difference was also noted between scores of the two professions for item 13 (*Z*_MW_ = −2.013; *p* = 0.044), item 14 (*Z*_MW_ = −2.021; *p* = 0.043) and item 16 (*Z*_MW_ = −3.399; *p* = 0.001).

**TABLE 2:** Mean (SD) scores for TIPI and mPPOS scores (*n* = 91).

Scale	Speech-language pathologist (*n* = 36)	Audiologist (*n* = 35)	All groups^d^ (*n* = 91)
**Ten Item Personality Inventory (TIPI) trait**			
Extraversion	4.6 (1.6)	4.7 (1.6)	4.6 (1.6)
Agreeableness	5.8 (1.1)	5.5 (1.0)	5.6 (1.0)
Conscientiousness	6.1 (1.0)	6.2 (0.8)	6.2 (0.9)
Emotional stability	4.7 (1.3)	4.9 (1.1)	4.8 (1.3)
Openness	5.5 (0.9)	5.3 (1.2)	5.4 (1.1)
**Modified patient practitioner orientation scale item**			
1. The clinician is the one who should decide what gets talked about during the visit	4.4 (0.9)	4.2 (1.3)	4.4 (1.1)
2. Although healthcare is less personal these days, this is a small price to pay for communication advances	4.5 (0.9)	4.3 (1.1)	4.5 (1.1)
3. The most important part of the standard appointment is the assessment	3.9 (1.3)	3.8 (1.2)	3.9 (1.3)
4. It is often best for clients if they do not have the full explanation of their communication impairment	5.6 (0.7)	5.4 (1.0)	5.5 (1.3)
5. Clients should rely on their practitioners knowledge and not try to find out about their conditions on their own	4.5 (1.2)	4.3 (1.5)	4.5 (1.3)
6. When practitioners ask a lot of questions about a client’s background, they are prying too much into personal matters	5.2 (0.8)	5.1 (1.0)	5.1 (1.1)
7. If practitioners are truly good at diagnosis and treatment, the way they relate to clients is not that important	5.4 (1.0)	5.4 (1.0)	5.4 (1.0)
8. Many clients continue asking questions even though they are not learning anything new	4.5 (1.1)	4.4 (1.1)	4.4 (1.2)
9. Clients should be treated as if they were partners with the clinician, equal in power and status^b^	5.1 (1.0)	4.8 (1.6)	4.9 (1.4)
**Sharing subscale**	**4.7 (0.5)**	**4.6 (0.8)**	**4.7 (0.7)**
11. If a clinician’s primary tools are being open and warm, the clinician will not have a lot of success	4.6 (1.4)	5.0 (1.0)	4.8 (1.2)
12. When clients disagree with their practitioners this is a sign that the clinician does not have the client’s respect and trust	4.1 (1.2)	4.1 (1.3)	4.3 (1.2)
13. A treatment plan cannot succeed if it is in conflict with the client’s lifestyle or values^b,^ ^c^	5.3 (0.9)	4.8 (1.1)	5.0 (1.1)
14. Most clients want to get in and out of the clinician’s office as quickly as possible^c^	4.5 (1.0)	3.9 (1.2)	4.2 (1.1)
15. The client must always be aware that the clinician is in charge	4.7 (1.2)	4.2 (1.3)	4.5 (1.2)
16. It is not that important to know a client’s culture and background in order to treat the client’s communication impairment^c^	5.8 (0.5)	5.2 (1.3)	5.4 (1.1)
18. When clients look up communication impairment information on their own, this usually confuses more than it helps	3.6 (1.0)	3.3 (1.1)	3.5 (1.1)
**Caring subscale** ^a^	**4.7 (0.5)**	**4.3 (0.6)**	**4.5 (0.7)**
**Total scale** ^a^	**4.7 (0.5)**	**4.5 (0.7)**	**4.6 (0.6)**

Score of 1 (strongly agree) = most clinician-centered; Score of 6 (strongly disagree) = most person-centered, bold items refer to the total scores of the included scales.

^a^Item 10 had a mean score of 3.39 (1.3) and item 17 (reversely scored) had a mean score of 3.8 (1.3 SD); they were both removed as reliability could not be established when they were added to the caring sub-scale.

^b^Items 9 and 13 are reversely worded items that were reverse scored.

^c^Significant differences were noted between SLP and Audiologist scores (Mann-Whitney, *p* < 0.05).

^d^All group includes SLP, Audiologists and practitioners practising in both fields.

Participants’ personality scores were highest in conscientiousness (6.2, 0.9 SD) and lowest in extroversion (4.6, 1.6 SD) across all practitioners (Table 2). There were no significant differences noted (*Z*_MW_ ranged from −1.351 to −0.148; *p*-values ranged from 0.177 to 0.882) across any of the personality traits between practitioner groups.

### 3.2. Predictors for PCC preference

Three robust quantile linear regression models for the total scale, sharing sub-scale and caring sub-scale were constructed, which revealed four common predictors (Table 3) across the mPPOS scores. All three models showed a reduction in percentage of error from the null model (no predictors) to the final model (significant predictors) of 10.3% (total), 13.9% (sharing), and 2.5% (caring).

**TABLE 3:** Significant predictors according to robust quantile regression analysis across mPPOS total, mPPOS sharing and caring subscales (*n* = 91).

Predictors	Coefficient (*ß*)	Std. error	*p*-value	95% confidence interval	
				**Lower bound**	**Upper bound**
**Total PPOS score intercept (excluding items 10 and 17)**	**4.450**	**0.206**	**<0.001**	**4.041**	**4.860**
Age^a^	0.015	0.005	0.007	0.004	0.026
10 other official South African Languages^b^	−0.414	0.123	0.001	−0.659	−0.169
Private sector^b^	−0.343	0.135	0.013	−0.611	−0.074
**Sharing scale intercept**	**4.514**	**0.227**	**0.000**	**4.063**	**4.965**
Age^a^	0.014	0.006	0.022	0.002	0.026
10 other official South African Languages^b^	−0.333	0.136	0.016	−0.603	−0.063
Academia^c^	−0.458	0.211	0.033	0.037	0.879
**Caring scale intercept (excluding items 10 and 17)**	**3.376**	**0.373**	**0.000**	**2.734**	**4.218**
Openness^a^	0.190	0.067	0.006	0.006	0.324

^a^Age and TIPI personality traits are continuous variables.

^b^Language had two categories with “English” as the benchmark.

^c^Sector had four categories with “public” as the benchmark.

Age was a significant predictor for the sharing subscale and the total mPPOS scale, indicating that for every one year increase in age, preferences toward PCC increased by 0.014 (*p* = 0.022) on the sharing mPPOS scores and 0.015 (*p* = 0.007) on the total mPPOS scores. If a participant’s home language was not English, it resulted in a significant decrease in mPPOS scores for the total (*ß* = −0.414, *p* = 0.001) and sharing subscale (*ß* = −0.333, *p* = 0.016) scores. Working in the private sector significantly decreased (*ß* = −0.343, *p* = 0.013) participants’ total mPPOS scores when compared to working in the public health sector and being in academia decreased sharing subscale scores (*ß* = −0.458, *p* = 0.033) when compared to being in the public sector. The personality trait of openness was the only trait significantly associated with an increase (*ß* = 0.190, *p* = 0.006) in mPPOS caring scores.

### 3.3. SLP/A views and understanding of PCC

Content analysis results of the open-ended question (*n* = 65) and focus group discussions (*n* = 9, 4 Audiologist, 3 Dual qualified, 2 Speech-language pathologists) revealed three categories: (i) Positive experiences with PCC (#151); (ii) Restrictions toward PCC (#63), and (iii) PCC exposure (#32) with 15 sub-categories (Supplementary Content 5). Supplementary Content 5 provides content analysis categories (*n* = 3), sub-categories (*n* = 13) and example quotations from open-ended question and focus group discussions.

The most common discussion points that emerged were related to positive experiences (#151) that practitioners mentioned as a result of providing care that was person-centered. The analysis resulted in four sub-categories, collaborative partnerships (#77), successful outcomes (#50), PCC an essential component (#14) and work satisfaction (#10). Secondly, restrictions toward providing PCC (#63) included practice setting (#15), time (#13), sociodemographic factors (#12), client restrictions (#12), professional buy-in (#7) and compassion fatigue (#4).

The third category that emerged was exposure to PCC (#32), where more recently qualified participants (<5 years) mentioned that they learnt about PCC at university (*n* = 8), with others mentioning that they completed IDA institute courses^1^ offered on PCC (#7). A few also indicated that they were exposed to it in their clinical setting (#7), CPD events (#4) or through their own personal experiences (#6).

## 4. Discussion

The study aimed to (i) identify preferences toward PCC; (ii) understand predictors of these preferences; and (iii) describe the understanding and views of PCC among SLP/As.

A high preference toward person-centeredness was noted across all groups (4.6, 0.6 SD) in this study. Although slightly higher than other studies, the mean scores in this study are comparable to mean scores of audiologists (4.46; 0.52 SD) and speech-language pathologists (4.07, 0.6 SD) practising in higher-income countries (Laplante-Lévesque et al., 2014; Bellon-Harn et al., 2017). In comparison to other diverse populations, the total mean scores (4.7, 0.5 SD) obtained in this study were much higher than those reported for audiologists across (3.6, 0.5 SD) culturally diverse populations which included in Iran, Portugal (3.4, 0.4 SD) and India (3.5, 0.5 SD) (Manchaiah et al., 2014). On comparison, participants in this study scored all items on each sub-scale higher than those involved in the study by Manchaiah et al. (2014). This studies results were also comparable to a recent meta-analysis on student practitioners across disciplines (4.16, 2.95 SD) (Bejarano et al., 2022). As noted in these studies, there was also a significant difference in the current study between the caring (4.5, 1.0 SD) and sharing (4.7, 0.7 SD) sub-scales, with the caring being significantly lower (*Z*_WSR_, = −2.778; *p* = 0.005) (Laplante-Lévesque et al., 2014; Bellon-Harn et al., 2017).

When comparing the scores obtained in this study between SLP and audiologists mean scores, most were similar with some variations. Speech-language pathologists scored significantly higher on items 13 (A treatment plan cannot succeed if it is in conflict with the client’s lifestyle or values), 14 (Most clients want to get in and out of the clinician’s office as quickly as possible), and 16 (It is not that important to know a client’s culture and background in order to treat the client’s communication impairment). The content of item 13 and item 14 may specifically relate to audiologists’ experiences with their diagnostic rather than rehabilitative roles (Manchaiah et al., 2017). Diagnostic audiological practices (i.e., diagnostic evaluations, routine hearing aid maintenance appointments) are mostly transient in nature when compared to the more long-term nature of speech-language therapy and enduring clinician-client relationships (Forsgren et al., 2022).

Cultural differences were noted in terms of practitioners’ home language not being English (*n* = 46) which negatively influenced practitioners’ preferences toward PCC (*ß* = −0.414, *p* = 0.001). Practitioners further acknowledged this during focus group discussions mentioning that sociodemographic factors (#12) are a barrier toward the implementation of PCC, specifically mentioning language and cultural diversity (“I do think that language and different cultures can be a barrier.”, Dual) Thus cultural differences should be acknowledged even in high-income western countries as there is a growing population of immigrants and refugees resulting in an increasing need to meet diverse populations healthcare needs (Ahmed et al., 2018).

In addition to home language, three additional factors, age, sector of employment (private/public/academic) and personality trait openness were found to be significant predictors toward PCC preferences. In terms of age, the current study found that older practitioners are more inclined toward PCC (*ß* = 0.015, *p* = 0.022). This is comparable to results from previous studies which indicate a positive association between age and preferences for person-centeredness (Krupat et al., 2000; Wahlqvist et al., 2010; Laplante-Lévesque et al., 2014). Increasing age and personal experience may result in practitioners developing a more nuanced perspective and deeper understanding of the value of PCC.

Working in the private healthcare sector negatively correlated to PCC. For practitioners who work in private sector settings, a lower preference for PCC was noted when compared to other employment settings (public sector and academia) (*ß* = −0.343, *p* = 0.013). This phenomenon was also noted by Laplante-Lévesque et al. (2014) in a group of Australian audiologists. The private sector may be more influenced by financial interests (e.g., the number of clients seen and hearing aid sales) that could potentially conflict with what is best for the client (Boisvert et al., 2017). The qualitative findings of the current study confirm that professional buy-in (#7), regardless of the context is important. One participant reported getting *“complaints from colleagues that I am taking longer” (Audiologist)*, whilst another participant highlighted that *“everyone in the team needs to buy in*…*to see the value” (Dual practitioner)*. Practitioners that are more person-centered may improve client satisfaction, which c ould ultimately lead to clients that remain loyal and recommending their clinician to others, thereby expanding the practice (Potter et al., 2003; Vonberg, 2022).

Interestingly, the two highest-scoring personality traits, conscientiousness (6.2, 0.9 SD) and agreeableness (5.6, 1.0 SD), did not influence practitioners’ preferences toward PCC. However, the personality trait openness (5.4. 1.1 SD), was a significant predictor of practitioners’ caring preferences. Practitioners scoring higher in openness were significantly (*p* < 0.05, coefficient 0.190) more likely to be imaginative, curious about other people and focused on tackling new challenges (Cherry, 2022). Out of the five personality traits, openness emphasizes insight the most (Cherry, 2022). This highlights the fact that insight into the communication challenges experienced by clients, to guide the development and implementation of individualized management plans is important.

The qualitative findings of this study further highlights practitioners’ positive experiences with PCC. Participants indicated that PCC is “*essential*” and a “*crucial part*” of intervention and, if implemented, it will result in “*client satisfaction*” and “*buy-in*” which can lead to “*improved generalisation*.” They also mentioned that PCC ensures a “*twofold interplay*” and allows the clinician to “*walk a mile in the patient’s shoes*” which facilitates “*improved outcomes*” and “*individualised care*.” This supports Forsgren et al. (2022) findings that effective partnerships between practitioners and clients are associated with listening carefully and focusing on the goals of their clients. Participants also mentioned that following a PCC approach has “*motivated*” them and it has been “*rewarding*” which results in overall “*work satisfaction*.”

Although participants acknowledged the value of implementing PCC, they felt that it “*takes a bit longer*” and they do not have the luxury of time due to “*large caseloads*.” A few participants also indicated that the diversity within the South African context results in a client population that is “*multilingual, multicultural*” making it challenging to provide PCC effectively. Contrary to experiencing work satisfaction (#10), a few participants mentioned that as they get invested in the client’s story, they experience “*compassion fatigue and burnout*” as they cannot draw “*professional boundaries*” and get too invested in “*their patient’s lives*.” Practitioners also experienced challenges as a result of their work setting, where some mentioned that being a speech-language pathologist in a school-setting makes it challenging to follow a PCC approach, whereas another mentioned professional buy-in also hinders being able to fully practise PCC as “*doctors just do not practise that way*.” It is clear that practitioners experience facilitators and barriers toward following a PCC approach which include personal experiences, resources, tools and workplace culture. These aspects require further investigation.

### 4.1. Study limitations

The study is not without limitations, generalization of the findings is hampered as findings represent a South African population as well as the small sample size, however, the gender distribution of the sample is representative of the gender profile of the professions where 95% are female (Pillay et al., 2020). Although a limited number of participants were involved in phase two, saturation of results was reached by the fourth focus group discussion (Gundumogula, 2020).

## 5. Conclusion

Speech-language pathologists and audiologists in South Africa tend to favor a PCC approach, although the outcomes are affected by certain factors, such as age and personality trait openness, as well as home language and sector of employment. The identification of these predictors highlights the need for a more comprehensive understanding of the barriers and facilitators that may influence the successful implementation of PCC in this context. To this end, future research should focus on investigating the macro, meso and micro systems that may impact the adoption of PCC by clinicians. Furthermore, a concerted effort should be made to expose practitioners to PCC through university training and continued professional development activities.

## Data availability statement

Data from this study are available from the corresponding author upon request.

## Ethics statement

The studies involving human participants were reviewed and approved by the Research and Ethics Committee, Faculty of Humanities, University of Pretoria, South Africa. The patients/participants provided their written informed consent to participate in this study.

## Author contributions

FM-A: conceptualization, methodology, investigation, data curation, writing original draft, and funding acquisition. LM: investigation and data curation. KJ and V-GH: writing review and editing. MG: formal analysis and data curation. DS: methodology and writing review and editing. All authors contributed to the article and approved the submitted version.

## Footnotes

1
https://idainstitute.com/tools/university_course/

## References

[B1] Ahmed S. Siad F. M. Manalili K. Lorenzetti D. L. Barbosa T. Lantion V. (2018). How to measure cultural competence when evaluating patient-centred care: A scoping review. *Br. Med. J. Open* 8 e021525. 10.1136/bmjopen-2018-021525 30018098 PMC6059336

[B2] Azkhosh M. Sahaf R. Rostami M. Ahmadi A. (2019). Reliability and validity of the 10-item personality inventory among older Iranians. *Psychol. Russia* 12 28–38. 10.11621/pir.2019.0303

[B3] Baicus C. Caraiola S. (2009). Effect measure for quantitative endpoints: Statistical versus clinical significance, or “how large the scale is? *Eur. J. Intern. Med.* 20 e124–e125. 10.1016/j.ejim.2008.10.002 19712834

[B4] Bejarano G. Csiernik B. Young J. J. Stuber K. Zadro J. R. (2022). Healthcare students’ attitudes towards patient centred care: a systematic review with meta-analysis. *BMC Med. Educ.* 22:324. 10.1186/s12909-022-03371-1 35477455 PMC9047330

[B5] Bellon-Harn M. L. Azios J. H. Dockens A. Manchaiah V. (2017). Speech-language pathologists’ preferences for patient-centeredness. *J. Commun. Disord.* 68 81–88. 10.1016/j.jcomdis.2017.06.012 28662420

[B6] Boisvert I. Clemesha J. Lundmark E. Crome E. Barr C. McMahon C. M. (2017). Decision-Making in Audiology: Balancing Evidence-Based Practice and Patient-Centered Care. *Trends Hear.* 2017:2331216517706397. 10.1177/2331216517706397 28752808 PMC5536381

[B7] Braun V. Clarke V. (2006). Using thematic analysis in psychology. *Qual. Res. Psychol*. 3, 77–101.

[B8] Cherry K. (2022). *The big five personality traits.* New York, NY: Very Well Mind.

[B9] Ehrhart M. G. Ehrhart K. H. Roesch S. C. Chung-Herrera B. G. Nadler K. Bradshaw K. (2009). Testing the latent factor structure and construct validity of the Ten-Item Personality Inventory. *Pers. Individ. Differ.* 47 900–905. 10.1016/j.paid.2009.07.012

[B10] Ekman I. Wolf A. Olsson L.-E. Taft C. Dudas K. Schaufelberger M. (2012). Effects of person-centred care in patients with chronic heart failure: The PCC-HF study. *Eur. Heart J.* 33 1112–1119. 10.1093/eurheartj/ehr306 21926072 PMC3751966

[B11] Elfstrand Corlin T. Kajonius P. J. Kazemi A. (2017). The impact of personality on person-centred care: A study of care staff in Swedish nursing homes. *Int. J. Older People Nurs.* 12:e12132. 10.1111/opn.12132 27696736

[B12] Entwistle V. A. Carter S. M. Cribb A. McCaffery K. (2010). Supporting patient autonomy: the importance of clinician–patient relationships. *J. Gener. Intern. Med.* 25 741–745. 10.1007/s11606-010-1292-2 20213206 PMC2881979

[B13] Faul F. Erdfelder E. Lang A.-G. Buchner A. (2007). G*Power 3: A flexible statistical power analysis program for the social, behavioral, and biomedical sciences. *Behav. Res. Methods* 39 175–191. 10.3758/BF03193146 17695343

[B14] Forsgren E. Åke S. Saldert C. (2022). ‘Person-centred care in speech-language therapy research and practice for adults: A scoping review. *Int. J. Lang. Commun. Disord.* 57 381–402. 10.1111/1460-6984.12690 34997806

[B15] Gosling S. D. Rentfrow P. J. Swann W. B. Jr. (2003). A very brief measure of the Big-Five personality domains. *J. Res. Pers.* 37 504–528. 10.1016/S0092-6566(03)00046-1

[B16] Grenness C. Hickson L. Laplante-Lévesque A. Davidson B. (2014). Person-centered care: A review for rehabilitative audiologists. *Int. J. Audiol.* 53 S60–S67. 10.3109/14992027.2013.866280 24447236

[B17] Gundumogula M. (2020). Importance of focus groups in qualitative research. *Int. J. Hum. Soc. Sci.* 8 299–302.

[B18] Håkansson Eklund J. Holmström I. K. Kumlin T. Kaminsky E. Skoglund K. Höglander J. (2019). Same same or different? A review of reviews of person-centered and patient-centered care. *Patient Educ. Counsel. J.* 102 3–11. 10.1016/j.pec.2018.08.029 30201221

[B19] Hanif A. (2018). Translation and validation of the Ten-Item Personality Inventory (TIPI) into Bahasa Indonesia. *Int. J. Res.* 7 59–69. 10.5861/ijrsp.2018.3009

[B20] Hofmans J. Kuppens P. Allik J. (2008). Is short in length short in content? An examination of the domain representation of the Ten Item Personality Inventory scales in Dutch language. *Pers. Individ. Differ.* 45 750–755. 10.1016/j.paid.2008.08.004

[B21] Jenkins D. G. Quintana-Ascencio P. F. (2020). A solution to minimum sample size for regressions. *PLoS One* 15:e0229345. 10.1371/journal.pone.0229345 32084211 PMC7034864

[B22] Krupat E. Hiam C. M. Fleming M. Z. Freeman P. (1999). Patient-centeredness and its correlates among first year medical students. *Int. J. Psychiatry Med.* 29 347–356. 10.2190/DVCQ-4LC8-NT7H-KE0L 10642908

[B23] Krupat E. Rosenkranz S. L. Yeager C. M. Barnard K. Putnam S. M. Inui T. S. (2000). The practice orientations of physicians and patients: The effect of doctor-patient congruence on satisfaction. *Patient Educ. Counsel.* 39 49–59. 10.1016/S0738-3991(99)00090-7 11013547

[B24] Laplante-Lévesque A. Hickson L. Grenness C. (2014). An Australian survey of audiologists’ preferences for patient-centeredness. *Int. J. Audiol.* 53 S76–S82. 10.3109/14992027.2013.832418 24447231

[B25] Leplege A. Gzil F. Cammelli M. Lefeve C. Pachoud B. Ville I. (2007). Person-centredness: conceptual and historical perspectives. *Disabil. Rehabil.* 29 1555–1565. 10.1080/09638280701618661 17922326

[B26] Lines L. Lepore M. Wiener W. (2015). Patient-centered, Person-centered, and Person-directed Care: They are Not the Same. *Med. Care* 53 561–563. 10.1097/MLR.0000000000000387 26067878

[B27] Manchaiah V. Dockens A. L. Bellon-Harn M. Burns E. S. (2017). Noncongruence between Audiologist and Patient Preferences for Patient-Centeredness. *J. Am. Acad. Audiol.* 28 636–643. 10.3766/jaaa.16084 28722646

[B28] Manchaiah V. Gomersall P. A. Tome D. Ahmed T. Krishna R. (2014). Audiologists’ preferences for patient centeredness: A cross-sectional questionnaire study of cross-cultural differences and similarities among professionals in Portugal, India, and Iran. *Br. Med. J. Open* 4:e005915. 10.1136/bmjopen-2014-005915 25763795 PMC4201997

[B29] Mead N. Bower P. (2000). Person-centeredness: A conceptual framework and review of emperical literature. *Soc. Sci. Med.* 51 1087–1110. 10.1016/S0277-9536(00)00098-8 11005395

[B30] Meyer C. Barr C. Khan A. Hickson L. (2017). Audiologist-patient communication profiles in hearing rehabilitation appointments. *Patient Educ. Counsel.* 100 1490–1498. 10.1016/j.pec.2017.03.022 28372897

[B31] Muck P. M. Hell B. Gosling S. D. (2007). Construct validation of a short five-factor model instrument. *Eur. J. Psychol. Assess.* 23 166–175.

[B32] Mudiyanse R. M. Pallegama R. W. Jayalath T. Dharmaratne S. Krupat E. (2015). Translation and validation of patient-practitioner orientation scale in Sri Lank. *Educ. Health* 28:35. 10.4103/1357-6283.161847 26261112

[B33] Nunes A. Limpo T. Lima C. F. Castro S. L. (2018). Short scales for the assessment of personality traits: Development and validation of the Portuguese Ten-Item Personality Inventory (TIPI). *Front. Psychol.* 9:461. 10.3389/fpsyg.2018.00461 29674989 PMC5895732

[B34] Onwuegbuzie A. J. Johnson R. B. Collins K. M. T. (2009). A call for mixed analysis: A philosophical framework for combining qualitative and quantitative. *Int. J. Multiple Res. Methods* 3 114–139.

[B35] Pallant J. (2020). *SPSS survival manual: A Step-by-Step Guide to Data Analysis Using IBM SPSS*, 7th Edn. London: Routledge.

[B36] Paul-Savoie E. Bourgault P. Gosselin E. Potvin S. Lafrenaye S. (2015). ‘Assessing patient-centered care: validation of the French Version of the Patient-Practitioner Orientation Scale (PPOS). *Eur. J. Person Center. Healthcare* 3 295–302.

[B37] Peeters M. J. (2016). Practical significance: Moving beyond statistical significance. *Curr. Pharmacy Teach. Learn.* 8 83–89. 10.1016/j.cptl.2015.09.001

[B38] Perestelo-Pérez L. Rivero-Santana A. González-González A. I. Bermejo-Caja C. J. Ramos-García V. Koatz D. (2021). Cross-cultural validation of the patient-practitioner orientation scale among primary care professionals in Spain. *Health Expect.* 24 33–41. 10.1111/hex.13135 33124759 PMC7879539

[B39] Pillay M. Tiwari R. Kathard H. Chikte U. (2020). Sustainable workforce: South African Audiologists and Speech Therapists. *Hum. Resour. Health* 18 1–13. 10.1186/s12960-020-00488-6 32611357 PMC7329495

[B40] Potter M. Gordon S. Hamer P. (2003). The physiotherapy experience in private practice: the patients’ perspective. *Austral. J. Physiother.* 49 195–202. 10.1016/S0004-9514(14)60239-7 12952519

[B41] Rogers C. R. (1951). *Client-centered therapy: Its current practice, implications, and theory*. Boston, MA: Houghton Mifflin Harcourt.

[B42] Romero E. Villar P. Gómez-Fraguela J. A. López-Romero L. (2012). Measuring personality traits with ultra-short scales: A study of the Ten Item Personality Inventory (TIPI) in a Spanish sample. *Pers. Individ. Differ.* 53 289–293. 10.1016/j.paid.2012.03.035

[B43] Santana M. J. Manalili K. Jolley R. J. Zelinsky S. Quan H. Lu M. (2018). How to practice person-centred care: A conceptual framework. *Health Expect.* 21 429–440. 10.1111/hex.12640 29151269 PMC5867327

[B44] Shi Z. Li S. Chen G. (2022). Assessing the psychometric properties of the Chinese version of Ten-Item Personality Inventory (TIPI) among medical college students. *Psychol. Res. Behav. Manage.* 15 1247–1258. 10.2147/PRBM.S357913 35603350 PMC9121988

[B45] Starfield B. (2011). Is patient-centered care the same as person-focused care? *Perman. J.* 15 63–69. 10.7812/tpp/10-148 21841928 PMC3140752

[B46] Storme M. Tavani J.-L. Myszkowski N. (2016). Psychometric properties of the French Ten-Item Personality Inventory (TIPI). *J. Individ. Differ.* 37 81–87. 10.1027/1614-0001/a000204

[B47] Téllez A. García C. H. Corral-Verdugo V. (2015). Effect size, confidence intervals and statistical power in psychological research. *Psychol. Russia* 8 27–46. 10.11621/pir.2015.0303

[B48] Vonberg J. (2022). How to transition your practice to person-centered care. *Hear. Rev.* 29 20–22.

[B49] Wahlqvist M. Gunnarsson R. K. Dahlgren G. Nordgren S. (2010). Patient centred attitudes among medical students: Gender and work experience in health care make a difference. *Med. Teach.* 32 191–198. 10.3109/01421591003657451 20353319

[B50] Watermeyer J. Kanji A. Cohen A. (2012). Caregiver recall and understanding of paediatric diagnostic information and assessment feedback. *Int. J. Audiol.* 51 864–869. 10.3109/14992027.2012.721014 23043520

[B51] Wisdom J. Creswell J. W. (2013). *Mixed methods: integrating quantitative and qualitative data collection and analysis while studying patient-centered medical home models.* Rockville, MD: Agency for Healthcare Research and Quality.

[B52] World Health Organization (2021). *World report on hearing.* Geneva: WHO.

