# Supplementary material for: Person-centered care: preferences and predictors in speech-language pathology and audiology practitioners
Source: Front Psychol. 2023 Jun 30;14:1162588. doi: 10.3389/fpsyg.2023.1162588 (PMC10348825; doi:10.3389/fpsyg.2023.1162588)
Supplement: Supplementary file 1 [file Data_Sheet_1.pdf]

Supplementary Content 1: e-Survey

The survey is made up of four short sections, a) biographic information, b) personality inventory c) modified Patient-Practitioner Orientation Scale and d) open ended question. Please complete all four sections.

Section A: Biographic information:

Please complete the information below by typing in or selecting the appropriate item from the dropdown menu:

Year of birth:_______________

Sex
- Male
- Female
- Prefer not to say
- Other (Specify)

Ethnicity
- White
- Indian
- African
- Asian
- Other (Specify)

Home Language
- English
- Afrikaans
- SeSotho
- isiZulu
- isiXhosa
- Sepedi
- Tswana
- Tsonga
- Swazi
- Venda
- Southern Ndebele
- Sign Language
- Other (Specify)

Based in:
- Gauteng
- North West
- Limpopo

- Eastern Cape
- KwaZulu Natal
- Mpumalanga
- Northern Cape
- North West
- Free State
- Other (Specify)

Currently practicing as:
- Speech-Language Pathologist
- Audiologist
- Dually qualified Speech- Language Pathologist and Audiologist

Sector:
- Private Practice
- Public sector
- Academia
- Other (Specify)

What was the most influential reason behind your career choice?
- An aptitude test result
- A family member with a communication or hearing disorder
- Career opportunities
- Expand knowledge base
- To help others
- Other (specify)

How many years have you been working in this field?

____

Client population serviced:
- 0 to 5 years old
- 6 to 18 years old
- 19 to 65 years old
- > 65 years old

Section B: Ten-Item Personality Inventory-(TIPI)

Below are a number of personality traits that may or may not apply to you. Please rank each statement to indicate the extent to which you agree or disagree with it. You should rate the extent to which the pair of traits applies to you, even if one characteristic applies more strongly than the other using the following 7 point likert scale:

I see myself as:

	1 Disagree Strongly	2 Disagree Moderately	3 Disagree a little	4 Neither agree or disagree	5 Agree a little	6 Agree Moderately	7 Agree Strongly
1.Extraverted, enthusiastic							
2. Critical, quarrelsome							
3.Dependable, self-disciplined							
4.Anxious, easily upset							
5. Open to new experiences, complex							
6. Reserved, quiet							
7. Sympathetic, warm							
8. Disorganized, careless							
9. Calm, emotionally stable							
10.Conventional, uncreative							

Section C: Modified Patient-Practioner Orientation Scale:
The term 'clinicians' refers to Audiologists and/or Speech-Language Pathologist. The term 'communication impairment' refers to a speech and/or audiology impairment.
For each item, rank your level of agreement with the statement using the six-point Likert scale:

	1 Strongly Agree	2 Agree	3 Slightly Agree	4 Slightly Disagree	5 Disagree	6 Strongly Disagree
1. The clinician is the one who should decide what gets talked about during the visit.						
2. Although health care is less personal these days, this is a small price to pay for communication advances.						

3. The most important part of the standard appointment is the assessment.						
4. It is often best for clients if they do not have the full explanation of their communication impairment.						
5. Clients should rely on their clinicians knowledge and not try to find out about their conditions on their own.						
6. When clinicians ask a lot of questions about a client's background, they are prying too much into personal matters.						
7. If clinicians are truly good at diagnosis and treatment, the way they relate to clients is not that important						
8. Many clients continue asking questions even though they are not learning anything new.						
9. Clients should be treated as if they were partners with the clinician, equal in power and status.						
10. Clients generally want reassurance rather than information about their communication impairment.						
11. If a clinician's primary tools are being open and warm, the clinician will not have a lot of success						
12. When clients disagree with their clinicians this is a sign that the clinician does not have the client's respect and trust.						
13. A treatment plan cannot succeed if it is in conflict with the client's lifestyle or values.						

14. Most clients want to get in and out of the clinician's office as quickly as possible.						
15. The client must always be aware that the clinicians is in charge.						
16. It is not that important to know a client's culture and background in order to treat the client's communication impairment.						
17. Humour is a major ingredient in the clinician's management of the client.						
18. When clients look up communication impairment information on their own, this usually confuses more than it helps.						

Section D: Open-ended question

We are interested in knowing what your personal opinion and views are about person-centered care. Please write 30 words or more in the space available below.

____

____

____

If you are willing to participate in a focus group, kindly provide your email address below so we can contact you.

Only if you leave your email addresses will you be approached to participate in the focus group. The virtual session will take place using Microsoft Teams at a suitable time for all participants and will not take longer than 45 minutes of your time. All participants who participate in the focus groups will receive a 1 GB data voucher.

____
