# Supplementary material for: Person-centered care: preferences and predictors in speech-language pathology and audiology practitioners
Source: Front Psychol. 2023 Jun 30;14:1162588. doi: 10.3389/fpsyg.2023.1162588 (PMC10348825; doi:10.3389/fpsyg.2023.1162588)
Supplement: Supplementary file 2 [file Data_Sheet_2.pdf]

Supplementary Content 2: Outline for focus group discussion

Welcome to today's discussion and thank you for taking the time to be here today. My name is xx and I am a xx at the xx University. I will be facilitating today's session. As you know, this study had two phases. Phase one entailed the completion of an online survey that you kindly completed. Phase two entails the 45 minute virtual session that we are currently busy with. Please note the session is being recorded to ensure that no comments are missed and assist during the transcription and analysis process.

Overview of the topic
Today we will be exploring your understanding and preferences towards person centered care.

Guidelines
There will be a few guidelines to facilitate this discussion today.

- The focus group should last no longer than 45 minutes.
- I will ask three questions that we will discuss as a group.
- Only one person should talk at a time, but everyone will be allowed to voice their opinions.
- Your honest feelings and opinions are what I would like to hear. You should not feel pressured as there are no right or wrong answers. I would just like to know your views even if they may be different from the other participants.
- Despite us all being on a first-name basis. All personal and identifiable data will be removed from the transcript to maintain confidentiality.

We have the following people who have joined us today: ………………..

Now that we all know more about each other. Let us begin with the discussion.

Opening Question
1. What is your understanding of Person Centered Care?

Note: A probe is only necessary if there is a lull in the discussion.

Free Probes	*Specific Probes*
• What else?<br>• Does anyone have a different thought?	• Where have you heard about person-centered care?<br>• What is your opinion on the objectives of Person centered care?

Approximate Time Allocation: 10 min

2. What do you feel are the limitations and benefits of utilizing a person centered approach?

Free Probes	*Specific Probes*
• What else?<br>• Does anyone have a different thought?<br>• Can you elaborate further?	• Why in your opinion do you think that is a benefit?<br>• Why in your opinion do you feel that is a limitation?

Approximate Time Allocation: 15 min

3. What content and exposure have you received to develop your skills in person-centered care?

Free Probes	*Specific Probes*
• What else?<br>• Does anyone have a different thought?<br>• Can you elaborate further?<br>• Can anyone think of anything else?	• Have you heard or discussed the topic at a conference or within your practice?<br>• Have you utilized a person centered approach during your clinical sessions?

Approximate Time Allocation: 15 min

That concludes our focus group discussion. Thank you so much for sharing your thoughts and opinions with us. A 1GB data voucher will be sent to each of you to cover the costs of the virtual discussion. Please send me an email with your service provider details.
