# Supplementary material for: Person-centered care: preferences and predictors in speech-language pathology and audiology practitioners
Source: Front Psychol. 2023 Jun 30;14:1162588. doi: 10.3389/fpsyg.2023.1162588 (PMC10348825; doi:10.3389/fpsyg.2023.1162588)
Supplement: Supplementary file 3 [file Data_Sheet_3.pdf]

Supplementary Content 3. Median (IQR) scores for Ten Item Personality Inventory (TIPI) and mPPOS scores (n=91)

Scale	Speech-language Pathologist (n=36)	Audiologist (n=35)	All Groups [a] (n=91)
**Ten Item Personality Inventory (TIPI) trait**			
Extraversion	4.5 (2.4)	5.0 (2.5)	4.5 (2.5)
Agreeableness	6.0 (1.5)	5.5 (2.0)	6.0 (1.5)
Conscientiousness	6.5 (1.5)	6.5 (0.5)	6.5 (1.5)
Emotional stability	4.8 (1.5)	5.0 (2.0)	5.0 (2.0)
Openness	5.8 (1.0)	5.5 (1.0)	5.5 (1.0)
**Modified Patient Practitioner Orientation Scale Item**			
1. The practitioner is the one who should decide what gets talked about during the visit	5.0 (1.0)	4.5 (2.0)	5.0 (1.8)
2. Although health care is less personal these days, this is a small price to pay for communication advances	4.0 (1.0)	5.0 (2.0)	5.0 (1.0)
3. The most important part of the standard appointment is the assessment	4.0 (2.0)	4.0 (2.0)	4.0 (2.0)
4. It is often best for clients if they do not have the full explanation of their communication impairment	6.0 (1.0)	6.0 (1.0)	6.0 (1.0)
5. Clients should rely on their practitioner knowledge and not try to find out about their conditions on their own	5.0 (1.0)	4.5 (3.0)	5.0 (1.0)
6. When practitioners ask a lot of questions about a client's background, they are prying too much into personal matters	5.0 (1.0)	5.0 (1.0)	5.0 (1.0)
7. If practitioners are truly good at diagnosis and treatment, the way they relate to clients is not that important	6.0 (1.0)	6.0 (1.0)	6.0 (1.0)
8. Many clients continue asking questions even though they are not learning anything new	5.0 (1.0)	5.0 (1.3)	5.0 (1.0)
9. Clients should be treated as if they were partners with the practitioners, equal in power and status [b]	5.0 (1.0)	5.0 (1.3)	5.0 (1.0)

**Sharing subscale**	**4.7 (0.8)**	**4.7 (1.1)**	**4.8 (0.7)**
11. If a practitioner's primary tools are being open and warm, the practitioners will not have a lot of success	5.0 (2.0)	5.0 (1.0)	5.0 (2.0)
12. When clients disagree with their practitioners this is a sign that the practitioners does not have the client's respect and trust	4.0 (2.0)	4.0 (2.0)	5.0 (0.2)
13. A treatment plan cannot succeed if it is in conflict with the client's lifestyle or values [b,c]	5.0 (1.0)	5.0 (2.0)	5.0 (1.0)
14. Most clients want to get in and out of the practitioner's office as quickly as possible [c]	5.0 (1.0)	4.0 (2.0)	4.0 (1.0)
15. The client must always be aware that the practitioners is in charge	5.0 (2.0)	4.0 (1.3)	5.0 (1.0)
16. It is not that important to know a client's culture and background in order to treat the client's communication impairment*	6.0 (0.0)	5.0 (1.0)	6.0 (1.0)
18. When clients look up communication impairment information on their own, this usually confuses more than it helps	4.0 (1.0)	3.0 (1.3)	3.0 (1.0)
**Caring subscale** [d]	**4.6 (0.9)**	**4.4 (0.6)**	**4.6 (0.8)**
**Total scale** [d]	**4.7 (0.8)**	**4.6 (0.7)**	**4.7 (0.6)**

Score of 1 (strongly agree)=most -practitioners-centered; Score of 6 (strongly disagree)=most patient-centered,
[a] All groups practitioners practicing as SLP, Audiologists or as both SLP and Audiologists
[b] Items 9, 13 are reversely worded items which were reverse scored.
[c] significant differences noted between SLP and Audiologist scores (Mann-Whitney, $p<0.05$)
[d] Item 10 had scores of 3.0 (2.0 IQR) and item 17 (reversely scored) had a score of 4.0 (1.0 IQR), they were both removed as inter-rater reliability could not be reached.
