# Supplementary material for: Person-centered care: preferences and predictors in speech-language pathology and audiology practitioners
Source: Front Psychol. 2023 Jun 30;14:1162588. doi: 10.3389/fpsyg.2023.1162588 (PMC10348825; doi:10.3389/fpsyg.2023.1162588)
Supplement: Supplementary file 4 [file Data_Sheet_4.pdf]

Supplementary Content 4: Inter-item correlations and corresponding p-values for TIPI and mean inter-item correlations and percentage of significant p-values for the mPPOS

**TIPI Scale**		
**Construct**	**rs**	**p-value**
Extroversion	0.673	$p<0.001$
Agreeableness	0.216	0.040
Conscientiousness	0.357	0.001
Emotional intelligence	0.474	$p<0.001$
Openness	0.251	0.017
**mPPOS scale**		
**Construct**	**Mean rs**	**% of significant p-values**
mPPOS Sharing	0.275	72.2%
mPPOS Caring (items 10 and 17 removed)	0.204	47.7%
Total mPPOS (items 10 and 17 removed)	0.224	53.3%
