# Supplementary material for: Person-centered care: preferences and predictors in speech-language pathology and audiology practitioners
Source: Front Psychol. 2023 Jun 30;14:1162588. doi: 10.3389/fpsyg.2023.1162588 (PMC10348825; doi:10.3389/fpsyg.2023.1162588)
Supplement: Supplementary file 5 [file Data_Sheet_5.pdf]

Supplementary Content 5. Content analysis categories (n=3), sub-categories (n=13) and example quotations from open-ended question and focus group discussions

Categories and sub-categories	Quotes
**Category 1: Positive experiences with PCC** (#151)	
**Collaborative partnership** (#77)	**"**I think person centered care is all about working towards co-production and co-creating … It is about giving the client the power to guide the clinician in ways that will help them benefit from services that they will receive. It helps in decision making and having them have their voices heard." (Audio)<br>"The patient is more engaged in the treatment program and better adapted to it." (Aud)<br>"Person-centered care is a way of involving the client and family members as part of treatment." (SLP)<br>**"**It is important for a patient to feel heard… this allows trust to develop and reassures that patient that the clinician has their best interests at heart." (Audio)<br>**"**It is vital that the client feels valued, heard and has an awareness of what is being worked on. A partnership where you guide but don't dictate and provide positive reinforcement allows for empowerment and success. Clients must feel special, comfortable and important." (SLP)<br>"So you're basically working hand in hand with the patient to provide services that are appropriate for them and that means you and them are sharing like the decisions in the services and you're cooperating to work on a better treatment for the patient themselves." (Aud)<br>"You know it's very much more slogan that's walk a mile in in someone else's shoes before you judge them that that's kind of, you know, there all sorts of variations on that, and I think, that in a way, the basis of what we're trying to achieve with person centered care." (Dual)<br>"So I think that it's a twofold interplay between the patient and the therapist when it comes to following person centered care". (Aud)
**Successful Outcomes** (#50)	"Clients are usually more satisfied with services rendered and more willing to return." (Aud)<br>"If we follow person centered care, the patient will tend to understand the therapeutic process, and we then hope to get buy-in from the patient, which ultimately should improve generalisation, and we hope that that would improve quality of life for the patient." (Audio)<br>"Without a patient feeling heard the therapy won't be a success." (SLP)<br>"Get the buy-in from them and hopefully aim towards generalization". (SLP)<br>"Basically working with the patient to get a better treatment plan out, because if they understand what's going to happen, then they're also more willing to participate". (Aud)
**Essential component** (#14)	**"**I believe that PCC is a crucial part to the work that we do." (Dual)<br>**"**PCC is essential for successful patient management." (Dual)<br>"PCC is the best way to provide services, particularly in a multi-lingual and cultural context. This model is recommended especially in the public sector. As a profession, we should advocate for the implementation of this approach to care." (Dual)

**Work Satisfaction** (#10)	"It's just rewarding in a sense that you can see the benefits of it for yourself as the therapist and as obviously for the client and the client's family." (Dual)<br>"I think seeing improvement in a patient's quality of life results in the clinician or me feeling like I've actually made a difference in their life and that I haven't wasted their time and that for me, is rewarding." (Dual)<br>"Very special and rewarding and as I said you have better work satisfaction." (Aud)<br>"I've actually made a difference in their life and that I haven't wasted their time and that for me, is rewarding." (Dual)
**Category 2: Restrictions towards PCC** (#63)	
**Practice setting** (#15)	"I think particularly in the state system, because the state system is so.. (exasperated sigh) regimented and so bullying of patients and patients have so little say" (Dual)<br>"I'm doing a lot of acute hospital work. And I mean some of the doctors, I mean, that's just not how they practice" (Dual)<br>"it's a huge thing, especially in public health, depending also on which province you are". (Aud)<br>"I do find it quite tricky in my situation when I'm working mainly at a school." (SLP)<br>"With POPI, it's just so difficult to get all the information from other professionals". (Dual)
**Time** (#13)	"I definitely think that some people feel that patient centered care might take a little bit longer… experienced it in my own practice as well." (Aud)<br>"Time, that was one of the main things that restrict me…I do get complaints from colleagues that I am taking a bit longer". (Aud)<br>"If we have larger caseloads, we don't have so much time to spend with patients specifically, patients who might have come from different backgrounds." (Dual)
**Socio-Demographics Factors** (#12)	"I do think that language and different cultures can be a barrier…We refer to multilingual and multicultural distinctive. I don't think we will ever be proficient, but I do think we can be sensitive." (Dual)<br>"Language barriers are my biggest challenge to practicing true PCC." (SLP)
**Client restrictions** (#12)	"But getting the family to buy in, I think regardless of whether it's an elderly person or a child. It's quite tricky to get the family committed to helping this person." (SLP)<br>"Patients can take offense or become very suspicious about why you're asking questions that are in a way none of your business …you know an invasion of their privacy." (Dual)
**Professional buy-in** (#7)	"I do get complaints from colleagues that I am taking a bit longer." (Aud)<br>" I think one of the things about PCC is that everyone in the team needs to buy in. Umm.. on that and…need to see the value of that." (Dual)<br>"so quite honestly it's not a term that comes up regularly in my practice or with any of the other people I work with" (Dual)<br>"With POPI, it's just so difficult to get all the information from other professionals" (Dual)
**Compassion Fatigue** (#4)	"Another thing that I've personally experienced definitely is compassion fatigue." (Aud)<br>"You tend to blur therapeutic boundaries." (Dual)<br>"I think compassion fatigue could be one of the limitations, like getting too involved in the therapeutic process". (Dual)
**Category 3: Exposure to PCC** (#32)	

**University Training** (#8)	**“**I haven't completed my community service yet, so everything about person centered care has mainly been from university. I think certain practical sites stress person centered, care more than others.” (SLP)<br>**“**PCC is something that I learned about at university, and I always said I will deliver a service that is PC because that is what I have learned but never really understanding what that means.” (SLP)
**IDA Institute** (#7)	**“**We have had training courses with the IDA Institute on Person Centered Care where we've had modules that we needed to complete.” (Aud)<br>**“T**hat little course from IDA I think we did in the 3rd or 4th year where we did inspired by IDA and then they somehow gave us the elements of patient centered care and how to go about it.” (SLP)
**Clinical Setting** (#7)	**“**This year I am beginning to understand what it means to deliver PCC as the population I am working with have different backgrounds, believes, cultures and languages and I have to take that into consideration.” (SLP)<br>**“**I have had exposure to PCC, but I have not implemented it in my practice to date.” (Dual)
**Personal Experience** (#6)	**“**I have never agreed with the pure medical model of service delivery as I have personal experience of the detrimental impact thereof.” (Dual)<br>**“**As a clinician and the parent of a child with complex communication needs, I know the importance of respect and genuine care given to the client and their families.” (Dual)
**Continuous Professional Development** (#4)	**“**I found that with a lot of the CPD activities for CPD points were always focused on this (Audt)<br>**“**[National Professional Association] talked the other week about decolonizing speech therapy, so I was quite interested in that because I kind of felt in a way that decolonizing and person centered care are, there's a kind of a mesh between the two in that.” (Dual)<br>**“**I'm part of a CPD journal club at the one hospital that I'm working… the psychologist in our team, the social worker and the occupational therapist, you know if I think about their presentations, I heard about it definitely from colleagues, not from speech therapy and audiology colleagues necessarily, but from multidisciplinary team members.” (Dual)

Dual refers to practitioners practicing as both SLP and Audiologists
frequency counts
